# Characterizing conformational states in GPCR structures using machine learning

**DOI:** 10.1038/s41598-023-47698-1

**Published:** 2024-01-11

**Authors:** Ilya Buyanov, Petr Popov

**Affiliations:** iMolecule, Skolkovo Institute of Science and Technology, Moscow, 121205 Russia

**Keywords:** Protein analysis, Protein function predictions

## Abstract

G protein-coupled receptors (GPCRs) play a pivotal role in signal transduction and represent attractive targets for drug development. Recent advances in structural biology have provided insights into GPCR conformational states, which are critical for understanding their signaling pathways and facilitating structure-based drug discovery. In this study, we introduce a machine learning approach for conformational state annotation of GPCRs. We represent GPCR conformations as high-dimensional feature vectors, incorporating information about amino acid residue pairs involved in the activation pathway. Using a dataset of GPCR conformations in inactive and active states obtained through molecular dynamics simulations, we trained machine learning models to distinguish between inactive-like and active-like conformations. The developed model provides interpretable predictions and can be used for the large-scale analysis of molecular dynamics trajectories of GPCRs.

## Introduction

G protein-coupled receptors (GPCRs) represent a large transmembrane protein family with more than 800 human genes discovered^1^. Their main function is signal transduction through different cellular pathways, mediated by heterotrimeric G proteins^2^ and β-arrestins^3^, that propagate downstream signal cascades^4–9^. Dysregulation in GPCRs signaling may lead to the development of various pathologies, including oncology^10^, cardiovascular diseases^11^, neurodegenerative diseases^12^, and many others. Therefore, GPCRs are one of the most important pharmacological targets, and nearly 35% of the approved drugs target the GPCR protein family^13^. Recent progress in crystallography^14^ and cryo-electron microscopy^15^ allowed the determination of the three-dimensional structures of GPCRs in complex with drugs or drug-like molecules, providing insight for the structure-based drug discovery. Structural studies revealed that GPCRs presumably reside in different conformational states, that can be roughly divided into active or inactive with respect to the signaling pathway^16^. Further analysis of ~230 structures of 45 class A GPCRs identified a group of 34 amino acid residue pairs that contribute to a common activation pathway^17^. These amino acid residues mainly correspond to the well-studied structural motifs across the transmembrane (TM) bundle, such as CWxP^18^, PIF^19^, sodium pocket^20^, NPxxY^21^ and DRY^22^). Remarkably, the structural differences between the active and inactive states can exceed several Angstroms in terms of the root-mean-square deviation (RMSD). These findings bring attention to the computer-aided structure-based drug discovery, where even subtle changes in the input structures may significantly affect the virtual ligand screening results^23^. Thus, the input conformations are of crucial importance and should be selected considering the target drug type, that is agonist or antagonist with respect to the signaling pathway. A possible solution is to use one or several experimental structures corresponding to the target drug type^24^; however, GPCR structure determination is a very difficult and resource-consuming problem^25^. Alternatively, one may use modeled structures that become more and more reliable with the progress in machine learning applied to protein folding^26,27^. However, state-of-the-art modeling approaches do not allow direct modeling of a GPCR in the target conformational state. Moreover, typically, one annotates a conformational state of a GPCR structure by visual inspection or using simple heuristics, such as measurements of the outward movement of the TM6 helix with respect to the TM core^28^. Due to the increasing number of GPCR structures corresponding to the active and inactive states, it became possible to apply the machine learning approaches for the classification of such GPCR structures^29^. Further, deep learning methods were developed for the classification of molecular dynamics trajectories of GPCRs^30^. To the best of our knowledge, the developed machine learning-based methods lack interpretability with respect to the known GPCR activation mechanism^17^. In this study, we present a machine learning approach for conformational state annotation of GPCRs, dubbed as STAGS (**ST**ate **A**nnotation of **G**pcr **S**tructures). We represent a GPCR conformation as a high dimensional feature vector comprising information about pairs of the amino acid residues contributing to the established GPCR activation pathway. Using 38 GPCR structures in inactive and active states, we constructed an annotated dataset of several thousand conformations obtained with molecular dynamic simulations. Next, we developed machine learning models to discriminate between inactive-like and active-like GPCR conformations. Finally, we demonstrated the use of the developed model in the downstream task, namely, the analysis of conformational ensembles obtained with molecular dynamics simulations.

## Results

### The STAGS workflow

**Figure 1:**
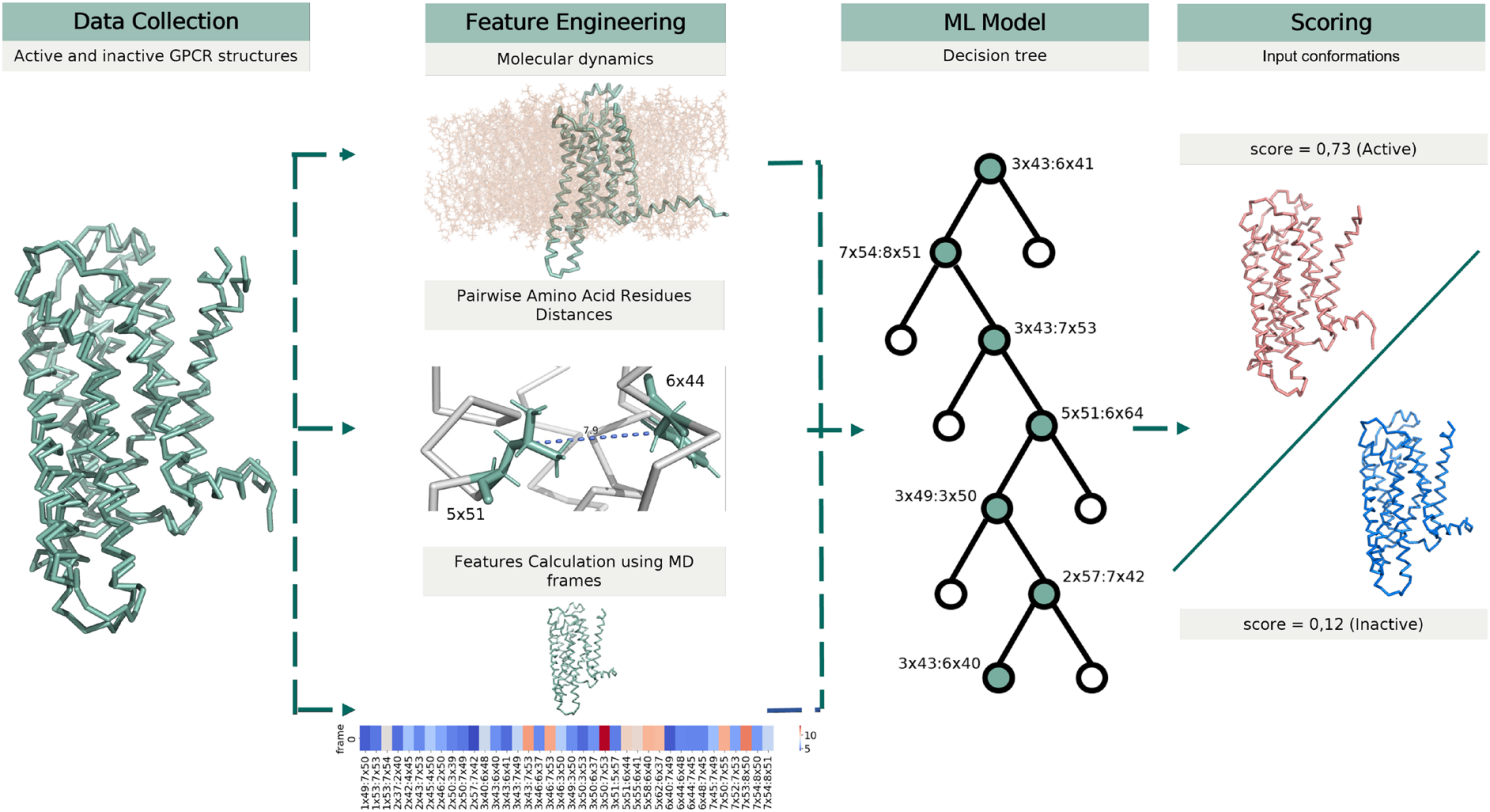
Overview of the STAGS workflow comprising the dataset collection, feature engineering, machine learning model training and application. *Data Collection * represents a composition of a dataset of active and inactive conformations of class A GPCRs. *Feature Engineering* illustrates a calculation of the descriptor corresponding to the pair-wise distances between amino acid residues from the molecular dynamics simulations. *ML Model* illustrates a decision tree from the random forest. *Scoring* demonstrates the calculated scores for active- and inactive-like conformations of a GPCR.

The developed machine learning model takes the high-dimensional representation of a GPCR structure as the input and outputs the probability of this structure to belong either to the active or inactive state, and Figure 1 illustrates the overview of the STAGS workflow. The high-dimensional representation comprises 38 pair-wise distances between the centers of amino acid residues side chains contributing to the common activation mechanism of class A GPCRs^17^. To train the STAGS model we collected a dataset of 10 active and 28 inactive crystallographic structures of Class A GPCRs (see Table S1), and run short-range (20 ns) full-atom molecular dynamics simulations for each structure. We then retrieved 200 conformations evenly distributed across the trajectories, resulting in $\sim 7,600$ structural models (see Methods), and calculated their high-dimensional representations, thus obtaining the $\sim 7,600 \times 38$ training matrix. Note, that longer simulations may result in the active to inactive conformational transitions^31^. Next, we derived machine learning models using stratified cluster-based splits for training and validation sets; we obtained the final model with nearly perfect accuracy for binary classification (see Table S2). Note, that decision trees commonly show state-of-the-art performance among classical machine learning approaches for tabular data, which is the case for structural motifs and microswitches in a GPCR^32^; moreover decision trees belong to the class of interpretable machine learning models^33^.

**Figure 2:**
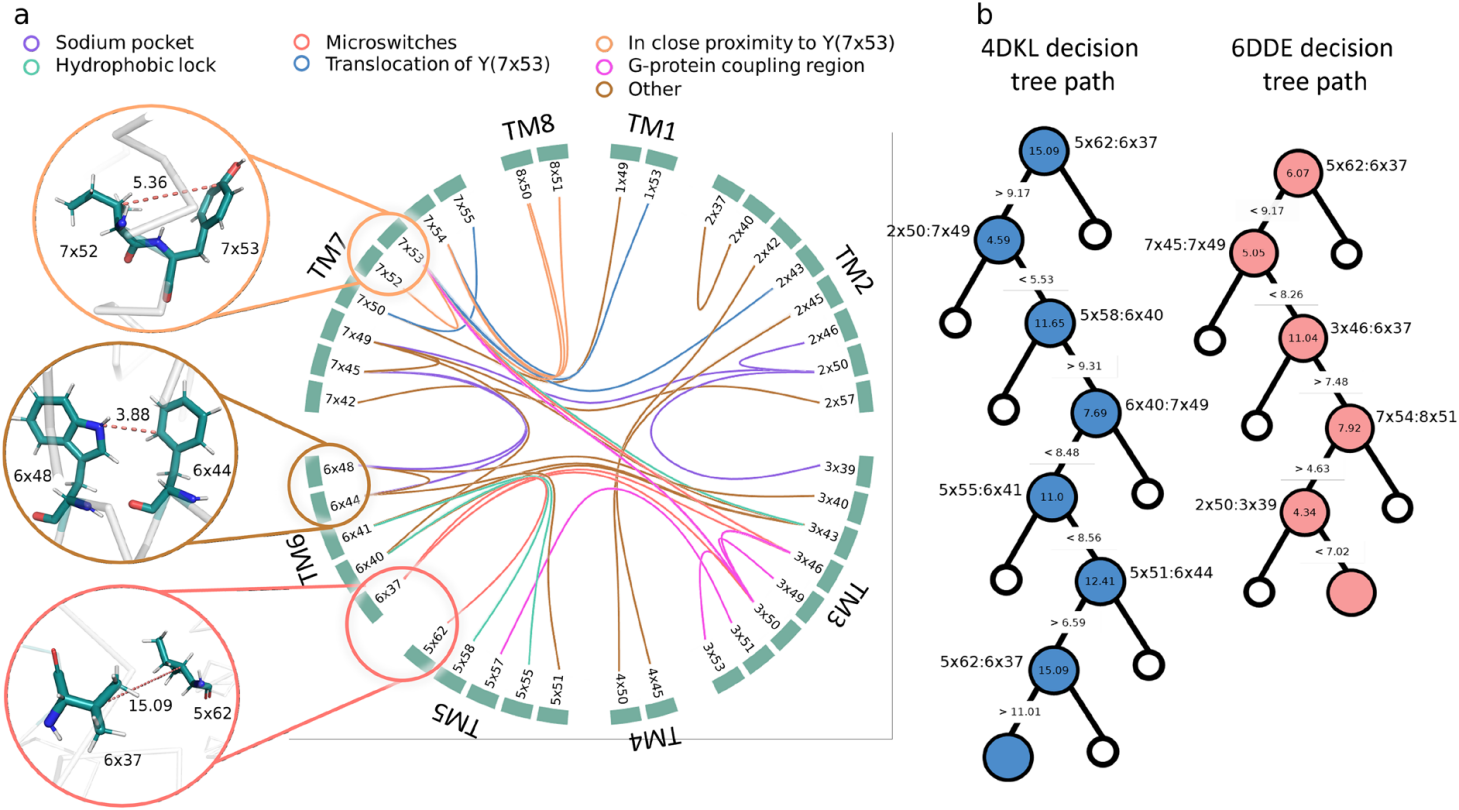
(**a**) A flare-plot highlighting pairs of residues (connected lines), that are used to calculate the features for the training set. Line colours correspond to the relation of a residue pair to the GPCR activation pathway. Three examples of pair-wise distances are shown for the μ-opioid receptor. (**b**) Illustration of a decision tree for the inactive-like (PDB ID:4DKL) and active-like (PDB ID:6DDE) structures of the μ-opioid receptor.

The STAGS interpretability makes it possible to understand the decision-making by traversing the model’s estimators. To demonstrate this, we considered the structures of μ-type opioid receptor in the active-like (PDB ID : 6DDE) and inactive-like (PDB ID : 4DKL) states (see Figure 2). In the case of the active-like state, a decision tree considers distances for residue pairs 5×62:6×37, 7×45:7×49, 3×46:6×37, 7×54:8×51, 2×50:3×39, while in the case of the inactive-like state, the decision tree considers distances for residue pairs 5×62:6×37, 2×50:7×49, 5×58:6×40, 6×40:7×49, 5×55:6×41, 5×51:6×44, according to the GPCRdb numbering scheme (see Methods).

These residues relate to microswitches (5×62:6×37, 3×46:6×37), hydrophobic lock (5×58:6×40, 5×55:6×41), sodium pocket (2×50:3×39, 2×50:7×49), and proximity to Y7×53 (7×54:8×51)^17^. The corresponding tree paths diverge on the 5×62:6×37 residue pair, because of the different distance between those residues (6.07 Å and 15.09 Å, for active- and inactive-like state structures, respectively), which corresponds to the mutual orientation between TM5 and TM6 important for the activation^34^. Interestingly, we observed that features, that are in the root of each estimator, typically involve residues of the transmembrane helix 6, for example 5×58:6×40, 3×46:6×37, and 5×62:6×37 (see Figure S4). Therefore, the STAGS model might capture the relative placement of the transmembrane helix 6 as one of the main characteristics of active/inactive GPCR states.

### Analysis of conformational ensembles

**Figure 3:**
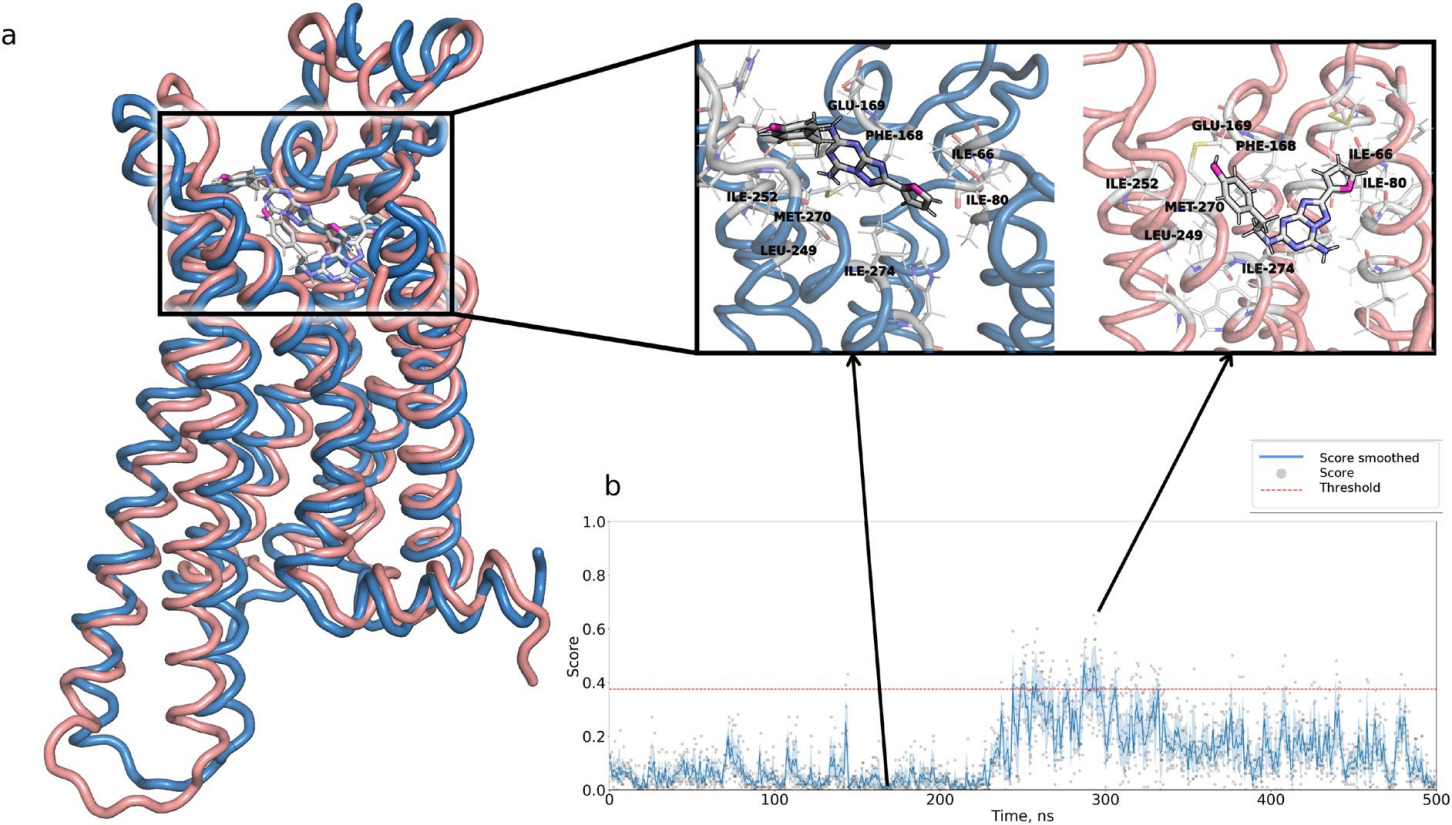
STAGS captures changes in receptor-ligand interactions across the molecular dynamics trajectory (GPCRmd ID: 4410464; starting structure PDB ID: 4EIY). (**a**) Snapshots of two conformations and ligand binding poses corresponding to the minimal and maximal STAGS scores. (**b**) STAGS’s score distribution across the molecular dynamics trajectory. The dots represent the score values, and the solid curve represents smoothed score over five consecutive frames. The red solid line corresponds to the STAG’s classification threshold.

For the first case study, we considered the molecular dynamics trajectory of the A2AR receptor in complex with the antagonist ZM241385 retrieved from GPCRmd^35^ (starting structure PDB ID: 4EIY). Figure 3b shows the trajectory profile with respect to the active state probability score obtained with STAGS. As one can see, most of the time the score is close to zero, indicating inactive-like conformations, which is expected. However, during ~0.1μs of the simulation, we observed a score increase, and for some frames, the score exceeds the active-like score threshold. A closer investigation of the MD trajectory revealed substantial changes in both the receptor conformation and the ligand binding pose (see Figure 3a). Indeed, when considering conformations *A* and *B* corresponding to the minimal and maximum scores of STAGS (0.0 and 0.65), the RMSD between the two ligand poses is 8.5 Å, so the receptor-ligand interactions differ a lot. For example, a non-polar contact between the furan ring of ZM241385 and ILE80(3×28) is present in the conformation *A*, but not the conformation *B* (4.0 Å vs. 8.4 Å); another example is a non-polar contact between the phenol group of ZM241385 and the oxygen atom of GLU169(5×30), which is stronger in conformation *B* compared to conformation *A* (1.7 Å vs. 3.1 Å). However, STAGS does not ’see’ the ligands and operates with the receptor conformations only; from this perspective, the model discriminates differences in pair-wise distances of residues related to the position of TM6 with respect to TM5 and TM7 (6×41:5×55 and 6×40:7×49). More precisely, the 6×41:5×55 distances are 8.78 Å and 4.9 Å and the 6×40:7×49 distances are 6.16 Å and 7.35 Å for the conformations *A* and *B*, respectively. Therefore, this case study demonstrates that STAGS can be used to detect important events in the molecular dynamics trajectories of GPCRs.

**Figure 4:**
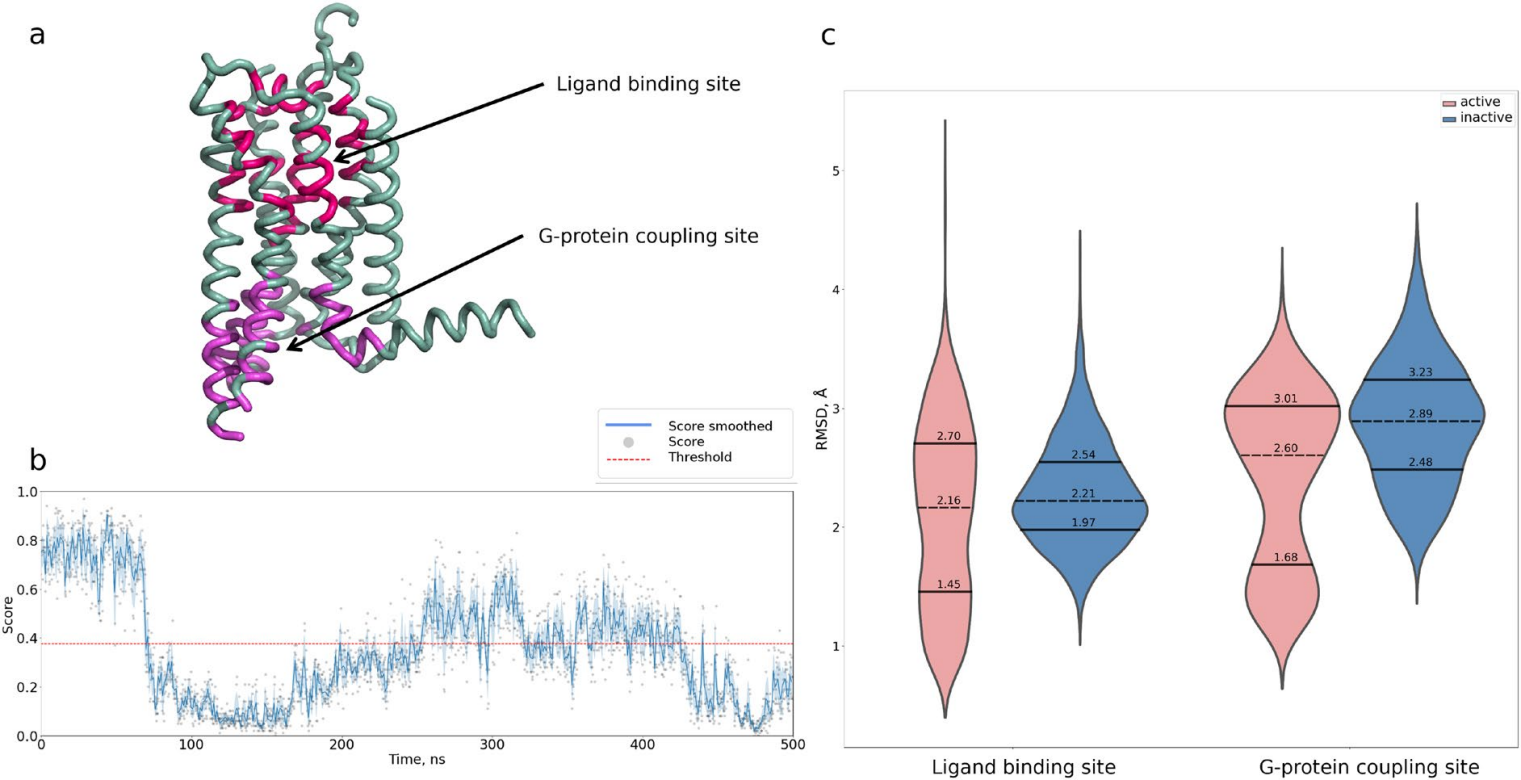
STAGS applied to the binding site analysis of the molecular dynamics trajectory of the GPCR intermediate state. (**a**) The structure of the A2a receptor (PDB ID: 5G53) with highlighted ligand (red) and G protein (magenta) binding sites. (**b**) The calculated STAGS’s score across the trajectory (GPCRmd ID: 3110356; starting structure PDB ID: 2YDO). (**c**) The distribution of the mean RMSD values for the subsets of trajectory frames classified as active (red) and inactive (blue).

To further explore STAGS applied to MD simulations, we considered a more complex trajectory started from the intermediate state structure (GPCRmd ID: $31-10356$; starting structure PDB ID: 2YDO). Accordingly, we observed a wide range of STAGS’s scores with $\sim 4:6$ ratio of active-to-inactive classified structures, given the score threshold of 0.375 (see Figure 4b). Interestingly, although the starting structure was characterized as intermediate^36^, it is in complex with the agonist adenosine; and the first part of the trajectory corresponds to the highest probability of active like conformations according to STAGS. Therefore, we hypothesized that STAGS might capture the binding site characteristics of the active/inactive structures of GPCRs. To test this hypothesis, we measured the binding site RMSDs between the MD frames and experimentally determined active GPCR structures, as it follows. We considered the ligand binding site as the residues within 8 Å from ligands in a set of active-like A2a structures (PDB IDs: 2YDO, 5WF5, 4UG2, 5G53, 3QAK, 4UHR, 2YDV, 6GDG, 5WF5, 5WF6, 7EZC). In addition, we considered the G protein binding site as the residues within 8 Å from the alpha helix of G$_{\alpha }$ subunit in the active A2a structure (PDB ID: 5G53) (see Figure 4a). Then, we calculated the RMSD of the binding sites between each frame and the selected structures after superimposition, and Figure 4c shows the obtained results. As one can see, for both ligand and G protein binding sites, the frames classified as active show smaller RMSD values compared to the frames classified as inactive. Indeed, the Mann-Whitney statistics p-values corresponding to the null hypothesis, that there are no differences between RMSD values for the frames classified as active and inactive, are $2.5e-7$ and $9.2e-47$ for the ligand and G protein binding sites, respectively ($3.7e-12$ and $3.4e-73$ for the student t-test statistics). It is important to note, that the median RMSD values are close to each other: 2.16 Å vs. 2.22 Å and 2.6 Å and 2.89 Å for the ligand and G protein binding sites, respectively. This can be explained by the fact, that the common activation pathway of GPCRs covers not only the binding site residues; hence other residues can play a decisive role in the frame’s classification. Overall, this case study demonstrates that STAGS can distinguish between active- and inactive-like GPCR structures and can be applied to trace conformational changes in the receptors.

With the progress in long-scale molecular dynamics simulations, it is important that the derived models are feasible for large-scale calculations. To test STAGS in the high-throughput setup, we applied it to the class A GPCR MD simulations retrieved from GPCRmd. In the first step, we retrieved 1417 trajectories from GPCRmd (as for 11.07.2022) for 502 GPCRmd entries comprising 254 unique PDB IDs. Filtering entries with distorted structures or without information about the receptor’s state resulted in 468, 98, and 115 trajectories annotated as inactive, active, and intermediate, respectively (see Table S5). In total, the 566 trajectories comprised $\sim 2,000,000$ conformations, and it took STAGS $\sim 1$ day on a desktop computer (NVIDIA GeForce GTX 1650, AMD$\circledR$ Ryzen 7 3800x 8-core processor $\times$ 16) to process them. As GPCRmd has a human-based annotation of the MD trajectories, we were interested in whether STAGS can be used to classify the whole trajectory as well. However, as we demonstrated above, a trajectory may contain both active-like and inactive-like frames, therefore its classification is not straightforward. To classify a trajectory as active or inactive, we introduced a threshold $\delta \in [0.0;1.0]$; if the ratio of frames classified as active is larger than $\delta$ then the trajectory is classified as active (otherwise, inactive). We observed that for $\delta \in [0.2; 0.8]$ the classification accuracy is $\sim 0.95$ and it is decreasing for the other values (see Figure S1). Therefore, although in general human-based classification matches with the STAGS’s classification of the GPCR trajectories, many trajectories comprise frames that STAGS considered with a different state, indicating potentially important events in the simulation.

## Conclusion

In this study, we presented STAGS (State Annotation of GPCR Structures), a novel machine learning approach for conformational state annotation of G protein-coupled receptors (GPCRs). By constructing a dataset comprising several thousand GPCR conformations obtained through molecular dynamics simulations, we successfully trained machine learning models to discriminate between inactive-like and active-like GPCR states. Leveraging information about amino acid residue pairs contributing to the established GPCR activation pathway, our models provide interpretable predictions, enhancing our understanding of GPCR conformational dynamics. We demonstrated STAGS efficacy in analyzing molecular dynamics trajectories and identifying events related to the ligand binding pose as well as active-inactive conformational changes. The STAGS source code is available at https://github.com/i-Molecule/gpcr-3D-annotation.

## Methods

### Dataset

In order to create a dataset of class A GPCR molecular dynamics trajectories, we formed a set of 38 structures, including 10 active state structures and 28 inactive state structures (Table S1) according to the literature, where crystallographical structures were presented. For each of the 38 structural models, we ran short molecular dynamics simulations (20 nanoseconds) and stored 200 frames corresponding to each 100 picoseconds snapshot. Thus, we obtained a dataset of $38 \times 200 = 7,600$ structural models with known conformational states. Finally, for each frame, we calculated a descriptor consisting of 38 pair-wise distances between the residues involved in the common activation mechanism of class A GPCRs (see Table S2). It is important to note that during molecular dynamics simulation, a receptor could shift from one state to another. It has been reported that conformational transition between the different states may occur on a scale of several hundred nanoseconds^37^. Therefore, we performed only short simulations of 20 nanoseconds, to minimize the risk of training set quality deterioration. To verify that there are no apparent conformational transitions, we measured the RMSD profile of a simulation trajectory with respect to the active- and inactive-like GPCR structures. Figure S3 shows the obtained results, as one can see (i) the RMSD profiles for active and inactive-like structures are clearly separated, and (ii) lower RMSD profiles correspond to the state of the starting GPCR structure.

### Machine learning

For machine learning algorithms we considered SVM and Random forest approaches from the python sklearn^38^ package, as well as the XGBoost approach from the xgboost package^39^. It is important to note, that random splitting of the dataset into the train-validation-test would result in a biased performance because very similar frames of the same receptor are likely shared between the splits. Therefore, we divided the dataset into 38 folds, such that the entire molecular dynamics trajectory belongs to a particular fold. Then we split the dataset into train and test partitions of 28 and 10 folds, respectively, and used 5-fold cross-validation using the train partition. We used precision, recall, F1-score, accuracy, and MCC as the performance metrics:

1 $$\begin{aligned}{} & {} Precision = \frac{TP}{TP+FP} \end{aligned}$$

2 $$\begin{aligned}{} & {} Recall = \frac{TP}{TP+FN} \end{aligned}$$

3 $$\begin{aligned}{} & {} F\text {-}score = \frac{2*precision*recall}{precision+recall} \end{aligned}$$

4 $$\begin{aligned}{} & {} Accuracy = \frac{TP+TN}{TP+TN+FP+FN} \end{aligned}$$

5 $$\begin{aligned}{} & {} MCC = \frac{TP*TN - FP*FN}{\sqrt{(TP+FP)(TP+FN)(TN+FP)(TN+FN)}} \end{aligned}$$

where, TN, TP, FP and FN represent true negatives, true positives, false positives and false negatives, respectively. We observed high performance in terms of all metrics (see Table S3).

### Molecular dynamics

Prior to MD system assembly, the membrane for simulations was built once, for all the protein structures, using a membrane builder tool from CHARMM GUI web-server^40^. The membrane consisted of a homogeneous bilayer of 210 phosphatidylcholine (POPC) lipids. Receptors were placed into the membrane using InflateGRO methodology^41^. Note, that because different GPCRs have different sizes, the amount of lipid molecules that were replaced by different receptors varied. All molecular dynamics simulations were carried out in GROMACS 2020.3 software package^42^ using the Leap-Frog integration algorithm with 2-fs intervals. The system was built under periodic boundary conditions (PBC) with the dimensions of the computational cell set to 9.06 × 9.1 x 12.37 nm. CHARMM36 force field^43^ was utilized with the water model TIP3P^44^. The system was neutralized and Na^+^, Cl^−^ ions were added up to ionic strength equal to 0.15 mol/L. Energy minimization was performed using the Steepest Descent algorithm in 50000 steps until the maximum force was less than 1000.0 kJ/mol/nm. Equilibration consisted of two subsequent simulations: 100-ps NVT ensemble (constant Number of particles, Volume, and Temperature) with the V-rescale thermostat^45^ mode with fixed positions of the atoms of the protein backbone for relaxation of the environment and 1000-ps NPT ensemble (constant Number of particles, Pressure, and Temperature) with the Parrinello-Rahman barostat^46^. The LINCS (LINear Constraint Solver) algorithm^47^ was utilized for restriction of all bonds and heavy atoms in the NVT and NPT stages. Following the equilibration, the 20-ns MD trajectory was calculated using the Parrinello-Raman barostat with preliminary annealing of individual parts of the system: the lipid bilayer, protein, and water with dissolved ions through two points from a temperature of 5 K to 315 K (Table 1). Pressure coupling was performed using time constant equal to 10.0 ps. Temperature coupling was performed using time constant equal to 0.1 ps. The Coulomb interactions were considered explicitly at small distances (up to 1.2 ns), and the long-range part of the potential was approximated by the Ewald summation method. To consider van der Waals interactions, a cutoff of 1.2 nm was used. For each system, one corresponding MD trajectory was calculated.

**Table 1:** Molecular dynamics simulations parameters.

Step	Time	Tcouple	Pcouple	Integrator
Energy minimization	50 000 steps	–	–	Steepest descent minimization
Nvt	100 ps	V-rescale	–	Leap-frog
Npt	1000 ps	Nose-Hoover	Parrinello-Rahman	Leap-frog
Molecular dynamics	20 ns	V-rescale	Parrinello-Rahman	Leap-frog

### Features

We considered pairs of interacting amino acid residues, involved in common GPCR activation pathway^17^, as features suitable for classification of the conformational states of GPCR structures (see Table S2). More precisely, we calculated the distances between the two residues as the distance between the geometric centers of the residue side chains. We performed standard feature analysis, including feature importance and cross-correlation, and the most important features appeared to be associated with the G protein coupling region and microswitch residues (3×43:6×41, 3×50:7×53, 3:46:6×37). The cross-correlation analysis has shown that there were no strongly correlated features in our dataset (Pearson correlation coefficient |*r*| < 0.9). We have used the GPCRdb numbering scheme to denote the residues^48^, which is an improved version of the sequence-based Ballesteros-Weinstein numbering scheme^49^. For each transmembrane helix, the most conserved residue is assigned to a number 50, and the other residues within the helix are numbered with respect to this residue; for example, a residue 3×46 is four residues before the most conserved residue of the transmembrane helix 3.

### Supplementary Information

Supplementary Information.

## Data Availability

The source code to derive and apply the STAGS model is freely available at https://github.com/i-Molecule/gpcr-3D-annotation.
