# Supplementary material for: Characterizing conformational states in GPCR structures using machine learning
Source: Sci Rep. 2024 Jan 11;14:1098. doi: 10.1038/s41598-023-47698-1 (PMC10784458; doi:10.1038/s41598-023-47698-1)
Supplement: Supplementary file 1 — Supplementary Information. [file 41598_2023_47698_MOESM1_ESM.pdf]

PDB ID	Conformational state	Reference
3V2Y	inactive	https://pubmed.ncbi.nlm.nih.gov/22344443/
3VW7	inactive	https://pubmed.ncbi.nlm.nih.gov/23222541/
4DJH	inactive	https://pubmed.ncbi.nlm.nih.gov/22437504/
4DKL	inactive	https://pubmed.ncbi.nlm.nih.gov/22437502/
4EIY	inactive	https://pubmed.ncbi.nlm.nih.gov/22798613/
4EJ4	inactive	https://pubmed.ncbi.nlm.nih.gov/22596164/
4NTJ	inactive	https://pubmed.ncbi.nlm.nih.gov/24670650/
4RWD	inactive	https://pubmed.ncbi.nlm.nih.gov/25686086/
4XNW	inactive	https://pubmed.ncbi.nlm.nih.gov/25822790/
4XT1	active	https://pubmed.ncbi.nlm.nih.gov/25745166/
4XT3	active	https://pubmed.ncbi.nlm.nih.gov/25745166/
4Z36	inactive	https://pubmed.ncbi.nlm.nih.gov/26091040/
4ZUD	inactive	https://pubmed.ncbi.nlm.nih.gov/26420482/
5DHH	inactive	https://pubmed.ncbi.nlm.nih.gov/26526853/
5G53	active	https://pubmed.ncbi.nlm.nih.gov/27462812/
5NJ6	inactive	https://pubmed.ncbi.nlm.nih.gov/28445455/
5T1A	inactive	https://pubmed.ncbi.nlm.nih.gov/27926736/
5UEN	inactive	https://pubmed.ncbi.nlm.nih.gov/28235198/
5UIW	inactive	https://pubmed.ncbi.nlm.nih.gov/28636951/
5VBL	inactive	https://pubmed.ncbi.nlm.nih.gov/28528775/
5XJM	active	https://pubmed.ncbi.nlm.nih.gov/29967536/
5XPR	inactive	https://pubmed.ncbi.nlm.nih.gov/28805809/
5XSZ	inactive	https://pubmed.ncbi.nlm.nih.gov/28792932/
5ZBQ	inactive	https://pubmed.ncbi.nlm.nih.gov/29670288/
5ZKQ	inactive	https://pubmed.ncbi.nlm.nih.gov/29808000/
6B73	active	https://pubmed.ncbi.nlm.nih.gov/29307491/
6C1R	inactive	https://pubmed.ncbi.nlm.nih.gov/29867214/
6D26	inactive	https://pubmed.ncbi.nlm.nih.gov/30220562/
6D9H	active	https://pubmed.ncbi.nlm.nih.gov/29925945/
6DDE	active	https://pubmed.ncbi.nlm.nih.gov/29899455/
6DO1	active	https://pubmed.ncbi.nlm.nih.gov/30639100/
6GDG	active	https://pubmed.ncbi.nlm.nih.gov/29726815/
6IIV	inactive	https://pubmed.ncbi.nlm.nih.gov/30510189/
6ME2	inactive	https://pubmed.ncbi.nlm.nih.gov/31019306/
6ME8	inactive	https://pubmed.ncbi.nlm.nih.gov/31019305/
6LFO	active	https://pubmed.ncbi.nlm.nih.gov/32610344/
6LFL	inactive	https://pubmed.ncbi.nlm.nih.gov/32610344/
6KO5	inactive	https://pubmed.ncbi.nlm.nih.gov/32814772/

Table S1. List of GPCR structures used to compose a training set.

Residue pair (GPCRdb nomenclature)	Description
1x49:7x50	hydrophobic lock
1x53:7x53	translocation of Y(7×53) away from residue at 2×43 upon GPCR activation
1x53:7x54	translocation of Y(7×53) away from residue at 2×43 upon GPCR activation
2x37:2x40	other
2x42:4x45	other
2x43:7x53	translocation of Y(7×53) away from residue at 2×43 upon GPCR activation
2x45:4x50	other
2x46:2x50	sodium pocket, hydrophobic lock
2x50:3x39	sodium pocket
2x50:7x49	sodium pocket
2x57:7x42	other
3x40:6x48	other
3x43:6x40	other
3x43:6x41	other
3x43:7x49	other
3x43:7x53	hydrophobic lock
3x46:6x37	microswitch residues
3x46:7x53	microswitch residues
3x46:3x50	G-protein coupling region
3x49:3x50	G-protein coupling region
3x50:3x53	G-protein coupling region
3x50:6x37	microswitch residues
3x50:7x53	G-protein coupling region
3x51:5x57	G-protein coupling region
5x51:6x44	other
5x55:6x41	hydrophobic lock
5x58:6x40	hydrophobic lock
5x62:6x37	microswitch residues
6x40:7x49	other
6x44:6x48	other
6x44:7x45	sodium pocket
6x48:7x45	sodium pocket
7x45:7x49	other
7x50:7x55	translocation of Y(7×53) away from residue at 2×43 upon GPCR activation
7x52:7x53	in close proximity to Y(7×53)
7x53:8x50	in close proximity to Y(7×53)
7x54:8x50	in close proximity to Y(7×53)
7x54:8x51	in close proximity to Y(7×53)

Table S2. List of residue pairs used to compute descriptors for the training set.

Algorithm	MCC f1	MCC f2	MCC f3	MCC f4	MCC f5	Mean MCC	Std
RF	0.993	0.978	0.334	1.0	0.944	0.850	0.258
SVM	0.930	0.665	0.625	0.942	0.987	0.830	0.152
XGBoost	0.987	0.953	0.333	0.998	0.869	0.828	0.251
Algorithm	Acc f1	Acc f2	Acc f3	Acc f4	Acc f5	Mean acc	Std
RF	0.997	0.998	0.75	1.0	0.99	0.947	0.099
SVM	0.972	0.879	0.868	0.977	0.995	0.938	0.053
XGBoost	0.995	0.98	0.75	0.999	0.947	0.934	0.094
Algorithm	Prec f1	Prec f2	Prec f3	Prec f4	Prec f5	Mean prec	Std
RF	0.99	0.992	0.5	1.0	1.0	0.897	0.198
SVM	0.9	0.96	0.94	1.0	1.0	0.96	0.038
XGBoost	0.98	0.96	0.5	0.998	0.99	0.886	0.19
Algorithm	Rec f1	Rec f2	Rec f3	Rec f4	Rec f5	Mean rec	Std
RF	1.0	0.995	0.5	1.0	0.93	0.88	0.194
SVM	1.0	0.54	0.5	0.92	0.983	0.788	0.221
XGBoost	1.0	0.97	0.5	1.0	0.82	0.86	0.19
Algorithm	F1_sc f1	F1_sc f2	F1_sc f3	F1_sc f4	F1_sc f5	Mean F1_sc	Std
RF	0.995	0.994	0.5	1.0	0.963	0.89	0.196
SVM	0.95	0.69	0.65	0.96	0.99	0.85	0.14
XGBoost	0.99	0.97	0.5	1.0	0.898	0.87	0.19

Table S3. Performance metrics obtained in cross-validation. The MCC, Accuracy, Precision, Recall, and F1-score metrics are listed for each fold, followed by the corresponding mean value and standard deviation.

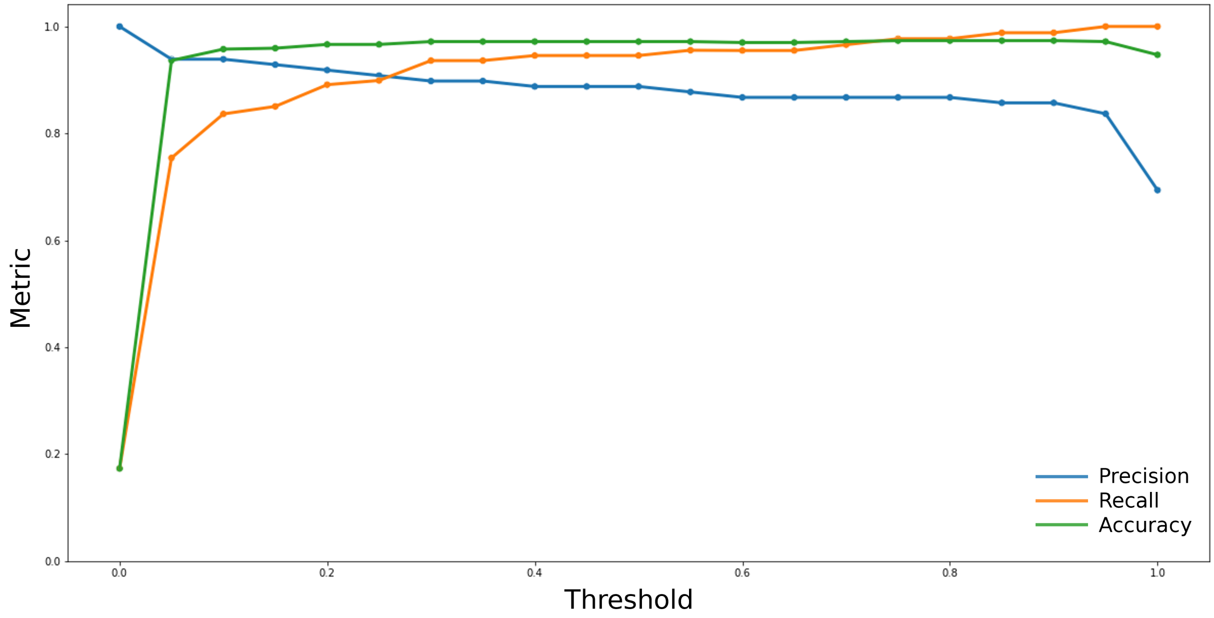

Figure S1. The Precision, Recall, and Accuracy metric values with respect to the classification threshold for the GPCRmd trajectory annotations.

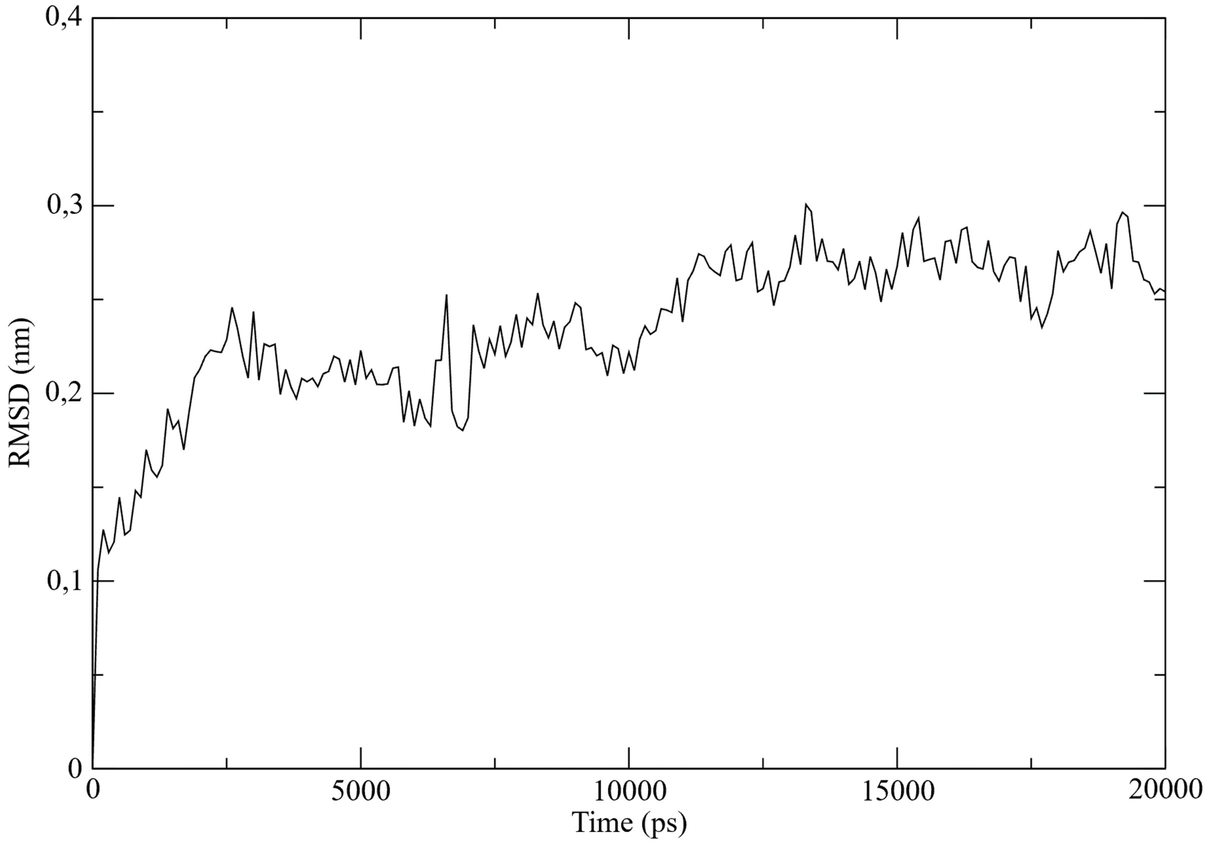

Figure S2. Example of the backbone RMSD values with respect to the starting conformation in a molecular dynamics trajectory.

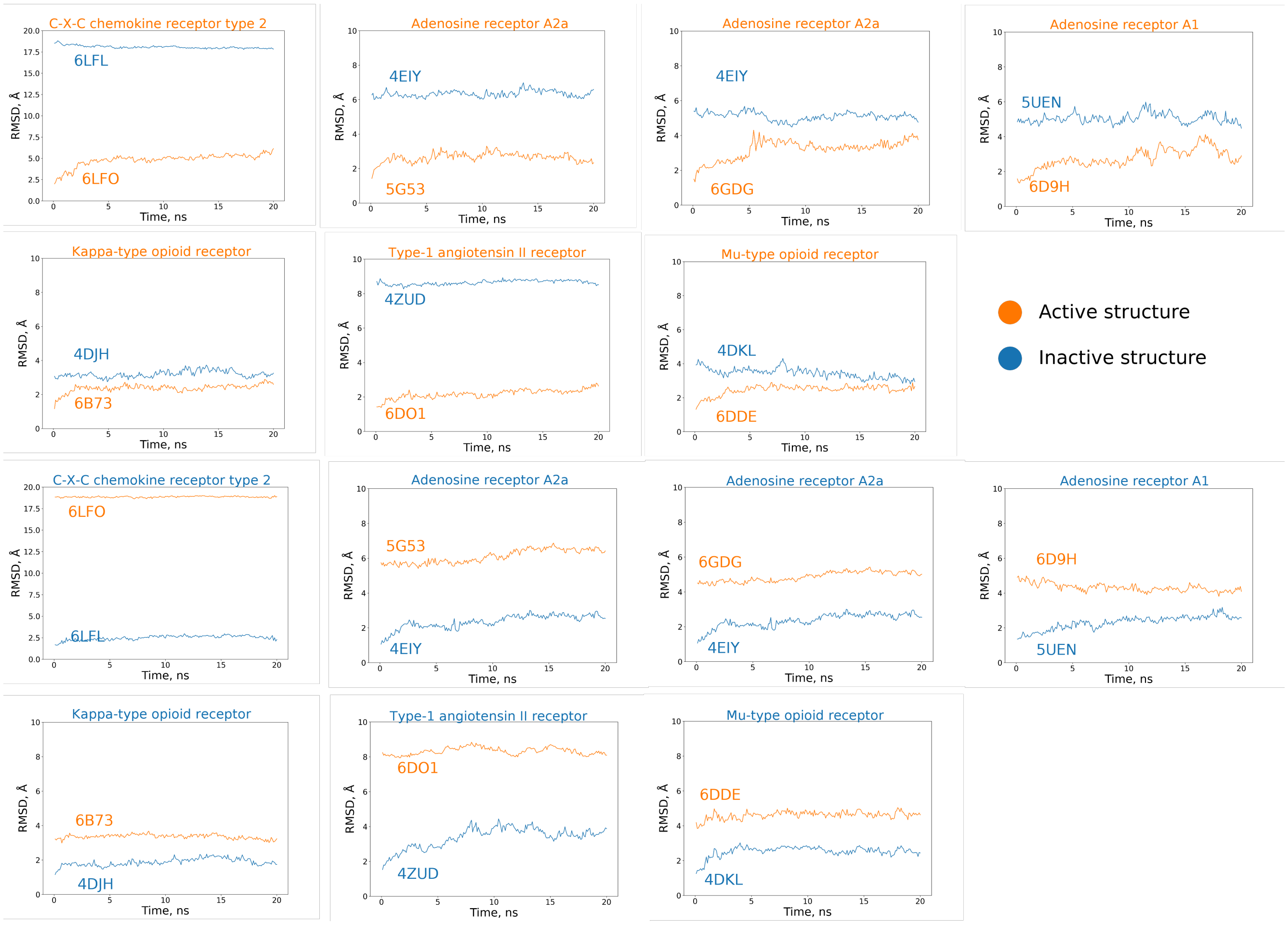

Figure S3. RMSD profiles of the molecular dynamic trajectories in the training set with respect to the active (orange) and inactive (blue) GPCR structures. The color of the RMSD profile corresponds to the starting GPCR structure, i.e. orange for active and blue for the inactive starting structure.

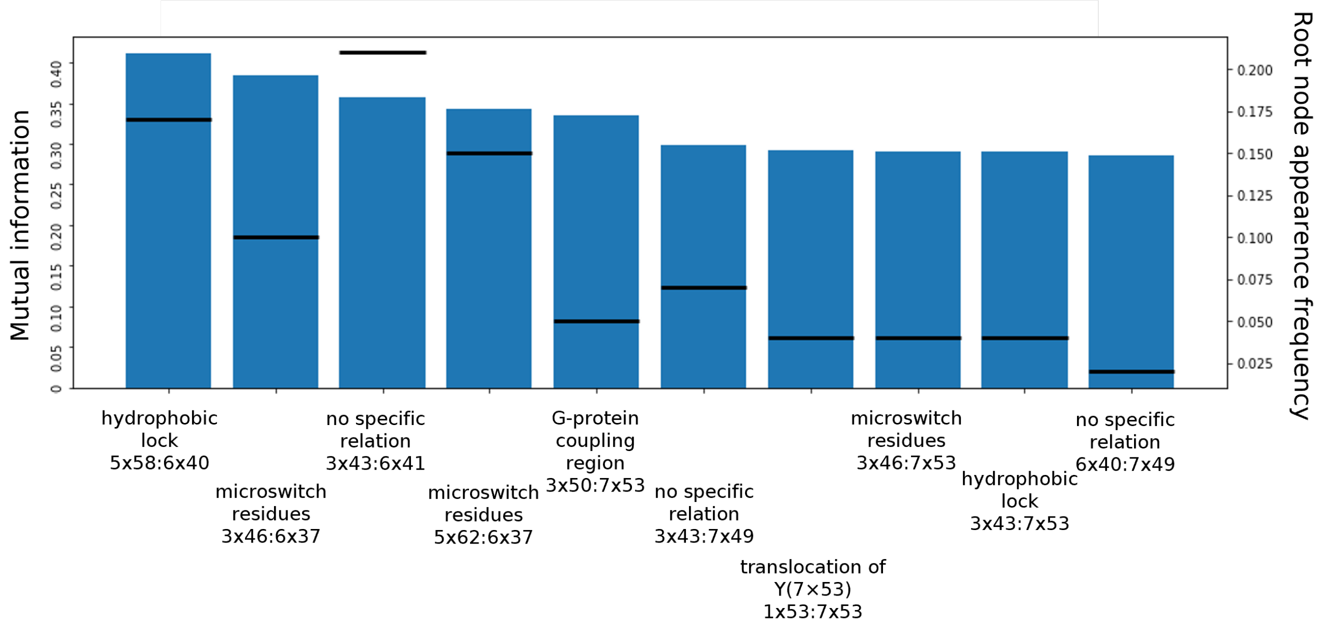

Figure S4. Top ten features in terms of the mutual information. The blue bars correspond to the mutual information calculated from the training data; the black horizontal lines correspond to the frequency of a feature observed in the root position of the decision trees.
